# Distinguishing Plasmablastic/Anaplastic Plasma Cell Neoplasms from Plasmablastic Lymphoma: A Narrative Review and Proposed Integrated Clinicopathologic Framework

**DOI:** 10.3390/life16101747

**Published:** 2026-10-18

**Authors:** Yini Alatan, Fenghao Zheng, Peiling Yu, Yinan Yuan, Hongyu Guo, Weilin Guo, Songwei Li, Shixuan Wang, Ailin Li, Siru Nie, Yuan Miao

**Affiliations:** 1 Department of Pathology, The First Affiliated Hospital and College of Basic Medical Sciences of China Medical University, Shenyang 110001, China; alatanyini@163.com (Y.A.); zhengfenghao0506@outlook.com (F.Z.); 2023110013@cmu.edu.cn (Y.Y.); guohongyu99999@163.com (H.G.); 15245396027@163.com (W.G.); 13504580535@163.com (S.L.); 15273637601@163.com (S.W.); 2 Department of Radiotherapy, Cancer Hospital of China Medical University, Liaoning Cancer Hospital and Institute, Cancer Hospital of Dalian University of Technology, Shenyang 110042, China; liailin0312@163.com

**Keywords:** plasmablastic plasma cell neoplasm, anaplastic plasma cell neoplasm, plasmablastic lymphoma, plasmablastic morphology, differential diagnosis

## Abstract

Plasmablastic/anaplastic plasma cell neoplasms (P/APC-N) and plasmablastic lymphoma (PBL) represent two categories of highly aggressive hematolymphoid malignancies. Their distinction is clinically important because they are classified and treated within different disease frameworks, yet no single morphologic, immunophenotypic, viral, cytogenetic, or molecular finding reliably resolves all cases. The proposed approach begins with the clinical and systemic context, including a pre-existing plasma cell neoplasm, marrow and serum findings, myeloma-defining events, immune status, and disease distribution. Morphologic assessment is followed by a panel evaluating plasma-cell differentiation, conventional B-cell markers, light-chain restriction, proliferative activity, and tumor-cell Epstein–Barr virus-encoded RNA (EBER) by in situ hybridization. Evidence of an established plasma cell neoplasm and a coherent myeloma-associated genomic background favor the relevant plasma cell neoplasm subtype, whereas tumor-cell EBER positivity in a compatible context supports PBL but is not specific. MYC protein overexpression, MYC rearrangement, high proliferative activity, light-chain restriction, and loss of conventional B-cell markers can occur in both categories and should not be used as stand-alone discriminators. When evidence does not converge, reports should state the findings favoring each category, identify unresolved conflicts, and recommend the next diagnostic steps. This framework remains to be validated in independent cohorts.

## 1. Introduction and Literature Search Strategy

In the 5th edition of the World Health Organization (WHO) Classification of Haematolymphoid Tumours, PBL is classified as a distinct disease within the large B-cell lymphoma family, defined as “an aggressive lymphoid neoplasm composed of large atypical B cells with plasmablastic or immunoblastic morphology, occurring primarily in extranodal sites and exhibiting a terminal B-cell differentiation phenotype” [1,2]. Plasma cell neoplasms with plasmablastic differentiation significantly overlap in their clinical presentation and histomorphology. Plasma cell neoplasms are a group of tumors characterized by the monoclonal proliferation of post-germinal center, terminally differentiated B cells that secrete monoclonal immunoglobulins. According to the 5th edition of the WHO Classification of Hematolymphoid Tumors, plasma cell neoplasms mainly include multiple myeloma (MM) and plasmacytoma. Although most plasma cell neoplasms are composed predominantly of mature plasma cells, a minority show plasmablastic or anaplastic morphology, which may cause diagnostic confusion with PBL [3]. For this review, we employ the author-defined umbrella term “plasmablastic/anaplastic plasma cell neoplasms” (P/APC-N) to collectively refer to plasma cell myeloma/multiple myeloma and plasmacytoma exhibiting plasmablastic or anaplastic morphology. P/APC-N should therefore be interpreted as descriptive shorthand used in this review and not as a distinct WHO-HAEM5 diagnostic entity. Within the spectrum of P/APC-N, a diagnosis of plasmablastic/anaplastic multiple myeloma (P/A-MM) first requires fulfillment of the diagnostic criteria for MM. This requires ≥10% clonal bone marrow plasma cells or a biopsy-proven bony or extramedullary plasmacytoma, together with at least one myeloma-defining event. These events comprise end-organ damage resulting from the plasma cell disorder, such as hypercalcemia, renal impairment, anemia, or osteolytic bone lesions (CRAB), or one of the SLiM biomarkers: ≥60% clonal bone marrow plasma cells; an involved-to-uninvolved serum free light-chain ratio ≥100, provided that the involved free light-chain concentration is ≥100 mg/L; or more than one focal lesion on magnetic resonance imaging, each measuring ≥5 mm. Building upon these conditions, the historical literature for plasmablastic diagnosis required plasmablasts to exceed 2% in bone marrow smears [4]. These historical systems were developed primarily for morphologic and prognostic stratification and should not be interpreted as current WHO-HAEM5 diagnostic criteria. Their thresholds are not interchangeable across bone marrow aspirate smears, core biopsies, and extramedullary tissue specimens. Accordingly, in this review, plasmablastic or anaplastic morphology is assessed descriptively and interpreted in conjunction with the formal diagnosis of the underlying plasma cell neoplasm and the integrated clinicopathologic findings. Plasmacytoma, including solitary plasmacytoma of bone (SPB) and extramedullary plasmacytoma (EMP), lacks evidence of MM or target organ damage. Regarding the plasmablastic type, Bartl et al. stated that a definitive diagnosis requires plasmablasts to constitute the predominant cell population within tumor infiltrative foci, without a specifically defined percentage [5]. Currently, no precise diagnostic threshold for the plasmablast proportion in plasma cell neoplasms has been established. PBL is a highly aggressive mature B-cell lymphoma characterized by a proliferation of large cells with plasmablastic or immunoblastic morphology, manifesting a terminal B-cell differentiation phenotype and primarily involving extranodal sites. It is categorized under large B-cell lymphomas. Histomorphologically, the tumor tissue must exhibit consistent plasmablastic or immunoblastic-like proliferation. Notably, the literature reports that a minority of PBL cases may contain tumor cells with plasmacytic morphology [1,6]. The immunophenotype must confirm plasma cell differentiation, characterized by strong positivity for core plasma cell transcription factors or surface antigens such as CD138, IRF4/MUM1, and PRDM1/BLIMP1. Because several other neoplasms may share plasmablastic morphology and a plasma-cell phenotype, alternative diagnoses should be excluded before the differential diagnosis is narrowed to PBL and P/APC-N [1]. A context-directed immunohistochemical panel should therefore be applied. This panel should include ALK immunohistochemistry to assess for ALK-positive large B-cell lymphoma [7], HHV8 immunohistochemistry to evaluate primary effusion lymphoma (PEL), its extracavitary variant, and other HHV8-associated large B-cell lymphomas [8,9], and extended B-cell lineage markers such as OCT2 and BOB1 when conventional B-cell markers are absent or equivocal [10]. CD30 and appropriate T-cell or cytotoxic markers should also be assessed when anaplastic large cell lymphoma is a diagnostic consideration [11]. In EBER-positive cases, Epstein–Barr virus (EBV) positive diffuse large B-cell lymphoma (DLBCL) should also be considered when the morphology and retained mature B-cell phenotype are not typical of PBL [12]. These findings must be interpreted together with morphology, disease distribution, immune status, and the relevant clinical context. The WHO recommends Epstein–Barr virus-encoded small RNA (EBER) in situ hybridization and molecular testing, as EBER positivity and fluorescence in situ hybridization (FISH)-confirmed *MYC* gene rearrangement are features supporting a PBL diagnosis. However, neither finding is specific or independently entity-defining because both can overlap with plasma cell neoplasms. Although monoclonal *IG* gene rearrangement serves as evidence of clonality, its value in differential diagnosis is limited as P/APC-N also exhibits this feature [1].

This narrative review was conducted based on a targeted evaluation of the literature relevant to distinguishing plasma cell neoplasms with plasmablastic or anaplastic morphology from plasmablastic lymphoma. Separate searches were performed using “anaplastic” with “multiple myeloma,” “plasmablastic” with “multiple myeloma,” “anaplastic” with “plasmacytoma,” and “plasmablastic” with “plasmacytoma,” as well as the term “plasmablastic lymphoma.” Relevant publications were identified through targeted literature searches and reference-list screening, with PubMed utilized for literature pertaining to plasmablastic and anaplastic plasma cell neoplasms. Publications were considered relevant if they addressed the classification or clinicopathologic distinction of plasma cell neoplasms with plasmablastic or anaplastic morphology and PBL. Publications unrelated to this diagnostic question were not used to support the diagnostic comparison; conventional MM and systemic EBV studies were retained where relevant for background context rather than entity-specific diagnostic performance. References were organized and managed using EndNote. The literature was updated through April 2026. The evidence hierarchy used in this review prioritized the WHO Classification of Hematolymphoid Tumors and other authoritative classification sources, peer-reviewed studies directly comparing the two diagnostic groups, larger clinicopathologic or molecular cohorts, and methodologically informative reviews. Small case series and individual case reports were used only to illustrate uncommon presentations and were not treated as reliable sources of diagnostic frequency or independent discriminatory weight.

## 2. Clinical and Biological Features

SPB accounts for approximately 70% of plasmacytomas and most often involves the axial skeleton. For EMP, approximately 80% of cases occur in the head and neck and upper respiratory tract, particularly the nasal cavity, paranasal sinuses, nasopharynx, and tonsils. Less common sites include the gastrointestinal tract, lungs, thyroid, and lymph nodes [1]. P/A-MM is associated with aggressive clinical behavior. Case reports have described cutaneous or central nervous system involvement, as well as malignant pleural or peritoneal effusions [13,14,15,16,17,18,19]. Furthermore, studies have reported that plasmablastic morphology is frequently associated with involvement of unusual anatomic sites, which can manifest as gastrointestinal involvement leading to massive upper gastrointestinal hemorrhage, or massive paraspinal masses causing severe spinal cord compression; an increased incidence of bone marrow fibrosis has also been observed [20,21,22]. In addition, following resistance to multiple lines of chemotherapy, patients with P/A-MM may develop the extremely rare complication of hyperammonemic encephalopathy, which leads to rapid mortality [21]. Progressive renal failure may also occur due to elevated plasma levels of free light chains [23].

PBL typically presents as extranodal masses, often in the oral cavity or gastrointestinal tract [1]. A review of the literature has summarized bone marrow involvement in approximately 40% of human immunodeficiency virus (HIV)-associated cases and approximately 25% of HIV-negative cases [24]. A separate Chinese series comprising 60 cases, in which all patients were HIV-negative, reported bone marrow involvement in approximately 49% [25]. Differences in cohort selection, staging, and case definitions limit direct comparison, and bone marrow involvement is not independently diagnostic.

When the clinical context is informative, a previous diagnosis of myeloma or plasmacytoma, or fulfillment of formal myeloma criteria, supports classification within the corresponding plasma cell neoplasm subtype. Significant immunodeficiency, including HIV infection, a post-transplant setting, or iatrogenic immunosuppression, may support PBL. Age, anatomic site, presenting symptoms, and bone marrow involvement overlap substantially and have limited independent discriminatory value. If the systemic context remains inconclusive, evaluation should proceed to morphology and lineage assessment.

Serum and urine monoclonal-protein studies provide important supportive context but are not entity-defining. In two recent real-world cohorts comprising 1622 Chinese and 12,582 US patients with newly diagnosed MM, IgG accounted for 48.4% and 59.1% of cases, IgA for 22.8% and 20.5%, light-chain-only disease for 20.4% and 19.3%, and IgM for only 0.43% and 0.70%, respectively [26]. In a selected 67-patient anaplastic MM cohort, IgG and IgA subtypes accounted for 31.3% and 25.4%, respectively, whereas nonsecretory disease accounted for 6.0% and no IgM cases were reported [27]. These distributions describe plasma cell neoplasm phenotypes rather than validated discriminators. A substantial serum or urine M protein, an abnormal involved-to-uninvolved free light-chain ratio, immunoparesis, or concordant marrow plasmacytosis favors a plasma cell neoplasm. By contrast, a serum paraprotein is uncommon in PBL (approximately 20% in compiled series), and reported monoclonal immunoglobulins in HIV-associated PBL have generally been below 20 g/L [28]. Cytoplasmic immunoglobulin is demonstrable in approximately 50–70% of PBL, but published series usually report light-chain restriction rather than reproducible IgG, IgA, and IgM heavy-chain distributions [28]. Therefore, no validated M-protein concentration or IgG, IgA, or IgM isotype cutoff distinguishes P/A-MM or plasmacytoma from PBL. Low or absent M protein does not exclude a plasma cell neoplasm, especially nonsecretory, oligosecretory, or extramedullary disease. Conversely, PBL may express cytoplasmic immunoglobulin and show light-chain restriction, and occasional patients have a detectable paraprotein [1,24,28,29]. These findings should be interpreted with myeloma-defining events, marrow findings, imaging, and the tissue phenotype.

## 3. Morphological Features

P/APC-N may contain varying proportions of plasma cells admixed with plasmablasts or anaplastic plasma cells, whereas PBL typically manifests as a destructive infiltrate of large immunoblastic and plasmablastic cells. Plasma cells show relatively mature plasmacytic morphology, characterized by eccentrically located round-to-oval nuclei, coarse clock-face chromatin, abundant basophilic cytoplasm, and a distinct perinuclear hof. Plasmablasts are characterized by large round-to-oval nuclei, finely dispersed or vesicular chromatin, a prominent centrally located nucleolus, and a high nuclear-to-cytoplasmic ratio. The cytoplasm is scant to moderate and basophilic, with an inconspicuous or absent perinuclear hof. Anaplastic plasma cells show marked cytologic pleomorphism and anaplasia, with considerable variation in cell size, irregular, folded, or multilobate nuclei, coarse-to-vesicular chromatin, prominent nucleoli, and occasional multinucleated or bizarre giant cells. Mitotic figures and apoptotic bodies are readily identified [1].

### 3.1. Shared Morphology

Both P/APC-N and PBL may contain plasmablasts. In rare cases, PBL can contain medium-sized lymphoplasmacytoid cells and plasma cells [1,6]. Although PBL may contain a plasmacytic component, this finding is uncommon [6]. Consequently, when tumor cells exhibit plasmacytic morphology, plasma cell neoplasms should remain the primary diagnostic consideration.

### 3.2. Potential Morphologic Clues and Limitations

**Figure 1:**
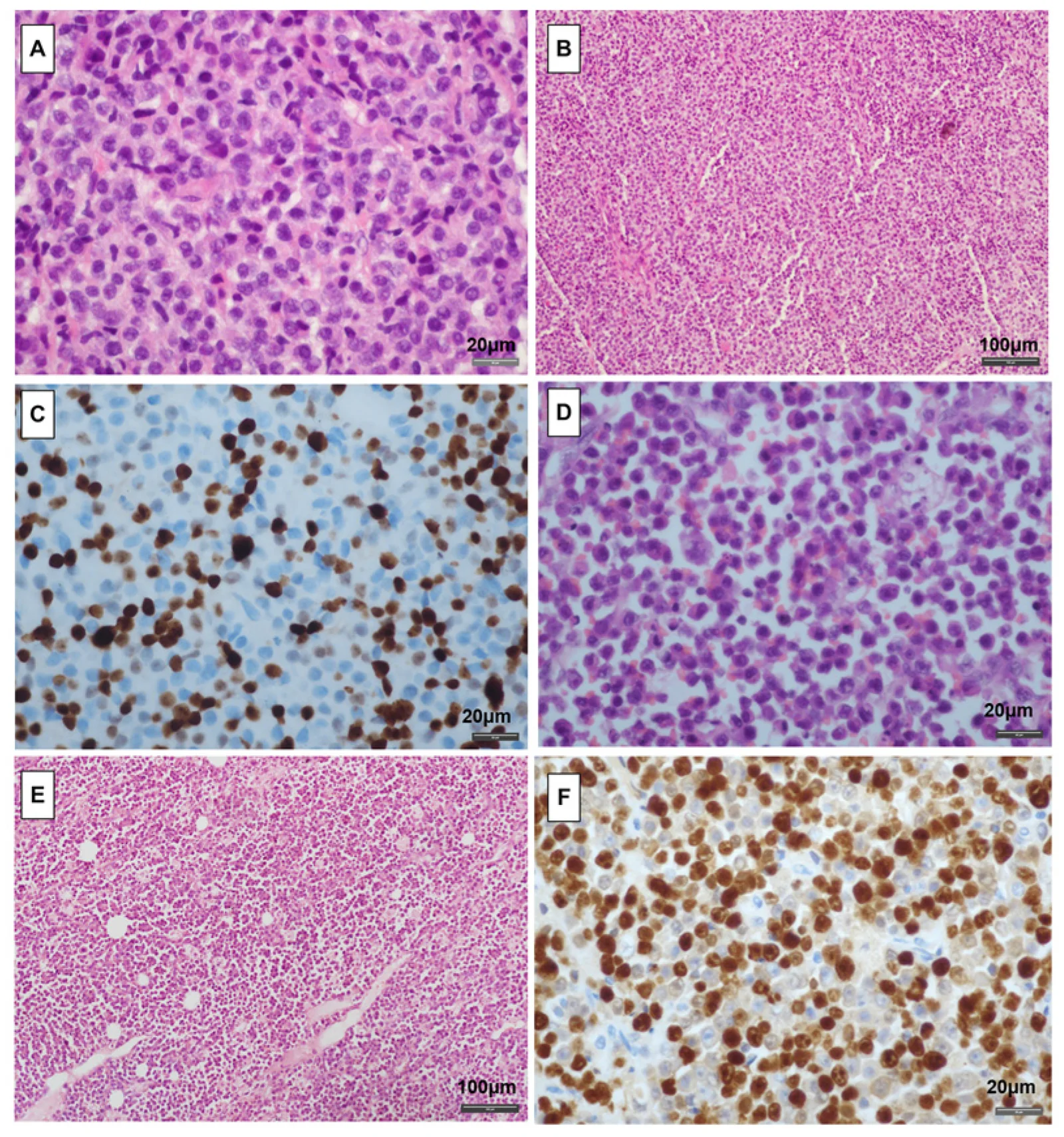
Histologic characteristics of plasmablastic plasmacytoma. (**A**–**F**) Representative findings from two cases. (**A**,**D**) Hematoxylin and eosin (H&E) staining shows sheets of large round-to-oval tumor cells with eccentrically located nuclei occupying approximately two-thirds of the cell volume and abundant cytoplasm. (**B**,**E**) H&E staining at lower magnification. (**C**,**F**) Ki-67 immunohistochemistry shows proliferation indices of approximately 45% in panel (**C**) and 80% in panel (**F**). Panels (**A**,**C**,**D**,**F**) are shown at an original magnification of ×400. Panels (**B**,**E**) are shown at an original magnification of ×100. Panels (**A**–**C**) and (**D**–**F**) were obtained from two different cases.

**Figure 2:**
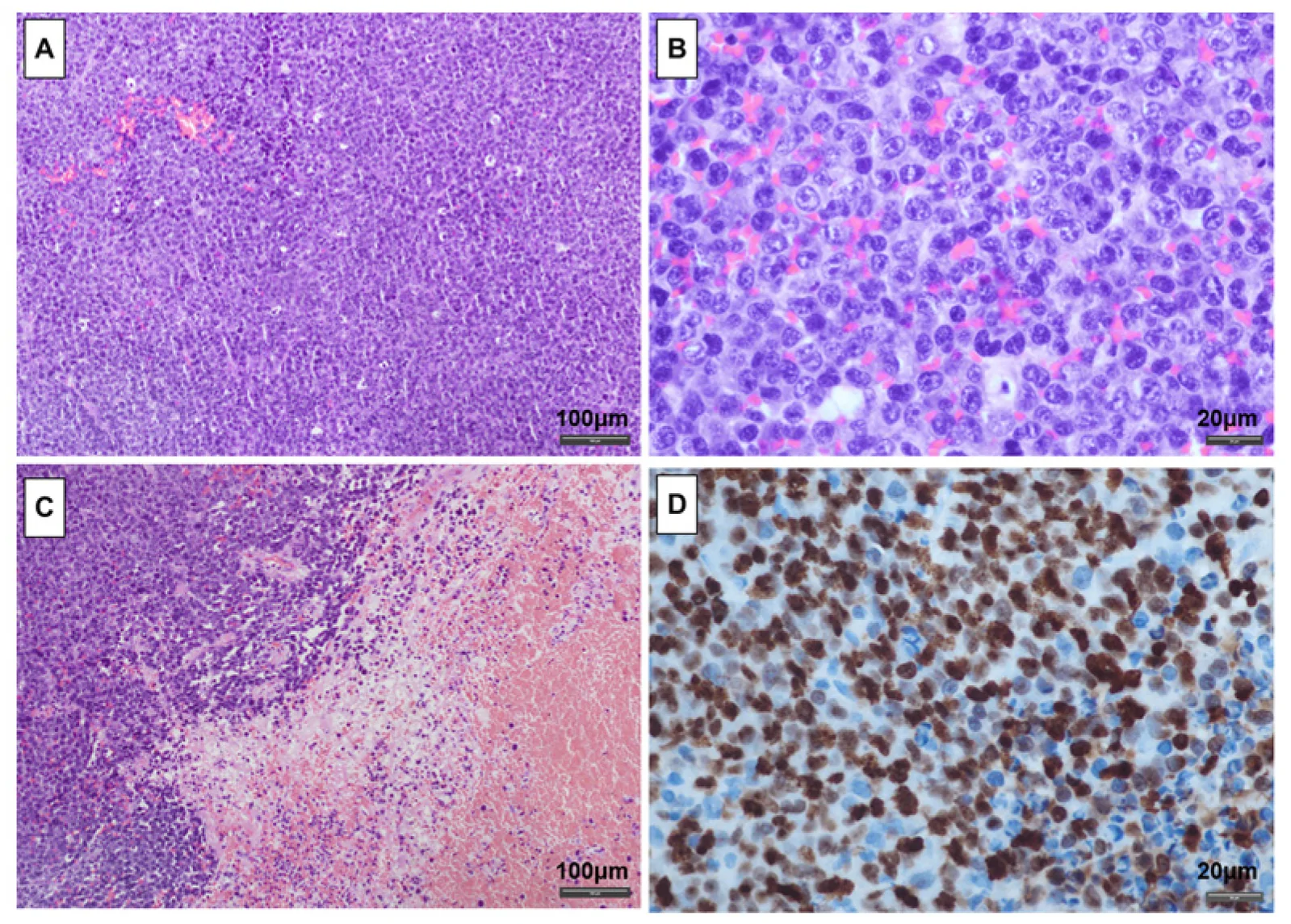
Histologic characteristics of plasmablastic lymphoma (PBL). (**A**) H&E staining shows a starry-sky pattern formed by macrophages containing apoptotic debris. (**B**) H&E staining shows large round-to-oval neoplastic cells with eccentrically located nuclei and a prominent central nucleolus. (**C**) Tumor necrosis is present. (**D**) Ki-67 immunohistochemistry shows a proliferation index of approximately 90%. Panels (**A**,**C**) are shown at an original magnification of ×100. Panels (**B**,**D**) are shown at an original magnification of ×400.

P/APC-N may contain varying proportions of plasmablasts and plasma cells, often with diffuse infiltration and interstitial fibrosis. A starry-sky pattern is uncommon (Figure 1), although tumor necrosis can occur [30]. PBL more often shows a diffuse destructive infiltrate. In one comparative study that included 49 PBL cases, a starry-sky pattern and tumor necrosis were reported in approximately 60% and 69% of cases, respectively [30] (Figure 2).

### 3.3. Diagnostic Pitfalls

Limited biopsy material may fail to represent the full morphologic spectrum of a lesion. A small sample may contain only the most plasmablastic area and omit residual cells with more mature plasma-cell differentiation or other architectural features that could help establish the disease context [28,31]. Necrosis, crush artifact, poor preservation, and consumption of tissue by multiple ancillary studies may further restrict interpretation. However, these features overlap and should not be used independently to classify an individual case. The tissue findings must also be correlated with previous diagnoses, bone marrow findings, serum or urine studies, imaging, and the systemic distribution of disease. When essential clinical information or adequate tissue is unavailable, the limitations of the specimen should be stated explicitly rather than concealed by a definitive classification.

When lymphoma or another plasmablastic neoplasm is suspected, fine-needle aspiration alone should not be relied upon for initial classification. Excisional or incisional biopsy is preferred when it can be obtained safely. If core needle biopsy is necessary, multiple cores should be collected and triaged prospectively for histology, immunohistochemistry, flow cytometry, tumor-cell EBER in situ hybridization, FISH, and molecular studies [31,32]. Prebiopsy PET/CT and cross-sectional imaging can help identify an accessible, metabolically active, viable site and avoid necrotic or previously treated tissue. If the initial sample is nondiagnostic or discordant with the clinical or imaging findings, repeat biopsy of a more representative site should be recommended [32].

## 4. Immunohistochemical Features

P/APC-N and PBL share a terminal B-cell/plasma-cell differentiation phenotype. Both entities commonly express CD38, CD138, IRF4/MUM1, and PRDM1/BLIMP1, with variable expression of XBP1. In contrast, mature B-cell markers such as CD20 and PAX5 are typically absent or only weakly expressed. IRF4/MUM1 serves as a single marker, whereas PRDM1/BLIMP1 is a distinct plasma-cell differentiation factor. CD79a may be retained in either entity and should not, by itself, be interpreted as evidence of a conventional mature B-cell phenotype. Accordingly, this shared immunophenotypic profile confirms terminal differentiation but generally does not distinguish P/APC-N from PBL. Kappa or lambda light-chain restriction supports the presence of a clonal B-cell or plasma-cell proliferation. However, it is common to both diagnostic categories and therefore has limited independent discriminatory value. Failure to demonstrate light-chain restriction does not exclude either diagnosis, particularly in small, necrotic, or poorly preserved specimens.

The immunophenotypic panel should therefore be interpreted based on diagnostic function. Plasma-cell differentiation markers establish lineage, light-chain studies assess clonality, and HHV8 helps exclude alternative HHV8-associated lymphomas. CD56, CD117, and cyclin D1 may provide contextually supportive evidence for a plasma cell neoplasm; however, their expression is variable and requires further study. Ki-67 and MYC protein expression reflect proliferative or oncogenic activity but overlap substantially between the two entities. MYC immunohistochemistry should not be considered equivalent to demonstration of a *MYC* rearrangement by FISH.

Accordingly, expression of CD38, CD138, IRF4/MUM1, and PRDM1/BLIMP1, in conjunction with absent or weak CD20 and PAX5, confirms terminal B-cell/plasma-cell differentiation but does not assign the case to PBL or a specific plasma cell neoplasm subtype [1].

Both categories frequently show light-chain restriction, and its frequency in PBL does not appear to depend on tumor-cell EBER status [33,34,35]. Extended markers may provide context, but no single immunohistochemical marker currently has sufficient discriminatory value to distinguish PBL from a plasma cell neoplasm with plasmablastic or anaplastic morphology (Table 1).

CD20, PAX5:

CD20 and PAX5 are usually absent in both categories, although weak or focal expression is documented in a minority of cases [28,36]. Their absence supports terminal differentiation but does not distinguish PBL from a plasma cell neoplasm.

CD45:

CD45 expression in PBL is variable and often weak or partial. Reported frequencies differ across cohorts and immune-status groups, with higher expression described in some post-transplant series [24,37].

In plasma cell neoplasms, CD45 is also variable and is usually absent or weak. Evidence specific to plasmablastic or anaplastic morphology is limited. One small series described acquisition of CD45 in two recurrent P/A-MM cases [38]. This isolated observation requires confirmation and has little independent diagnostic value.

CD56:

In PBL, the overall expression rate of CD56 is approximately 20% (5/24) [29,39]. Its expression level is correlated with the degree of histological differentiation: the CD56 positivity rate in PBL with distinct mature plasmablastic differentiation features is significantly higher than in cases presenting with monomorphic immunoblastic-like morphology (36% (5/14) vs. 9% (2/23), with an overall rate of approximately 19% (7/37)) [40]. In MM, CD56 is predominantly expressed by bone marrow plasma cells, with a positivity rate of 60–75% [41]. In contrast, the CD56 positivity rate in extramedullary plasmacytomas is exceedingly low, approximately 10% (3/28), with the majority of cases exhibiting only weak or focal expression [42]. Furthermore, a study showed that the CD56 positivity rate in P/A-MM is 56% (14/25) [43], while in EBV-positive anaplastic plasma cell myeloma, the CD56 positivity rate is also approximately 69.2% (9/13) [44].

CD81:

In MM, CD81 is predominantly expressed by bone marrow plasma cells, with a positivity rate of 40–50% [41]; research has found that CD81 positivity is associated with poor prognosis in plasma cell neoplasms [45], though no studies have investigated the relationship between CD81 and morphology. PBL generally does not express CD81 [28].

CD27:

The CD27 expression profile in P/APC-N remains to be further elucidated. In MM, CD27 is predominantly expressed by bone marrow plasma cells, with a positivity rate of 40–60% [41]. Conversely, the absence of CD27 expression in MM has been linked to an inferior clinical outcome [46]. Its relationship with plasmablastic morphology remains unclear. Genetic alterations involving *CD27* have been described in EBV-positive PBL [47], but current evidence does not support CD27 as a stand-alone diagnostic marker.

CD117:

PBL usually lacks CD117, although rare positive cases have been reported and results may depend on antibody clone and detection sensitivity [48,49]. In MM, CD117 is predominantly expressed by bone marrow plasma cells, with a positivity rate of 30–32% [41], but one recent study reported no CD117-positive cases in P/A-MM (*n* = 25) [43]. The available evidence is too limited for CD117 to serve as an independent discriminator.

Cyclin D1:

Cyclin D1 is usually absent in PBL, although rare positive cases occur. In conventional MM cohorts, cyclin D1 is expressed in a substantial subset, approximately 70% (14/20) [50,51], whereas the positivity rate of cyclin D1 in P/A-MM is approximately 16% (4/25) [43]. Cyclin D1 may support a myeloma-associated background when concordant with clinical and genetic findings, but its absence does not exclude a plasma cell neoplasm.

MYC protein expression:

MYC protein overexpression by immunohistochemistry, *MYC* rearrangement demonstrated by FISH or sequencing, and broader MYC pathway dysregulation represent distinct findings. In one P/A-MM cohort, MYC protein expression was detected in 17 of 23 tested cases (74%), whereas a *MYC* rearrangement was identified in 2 of 13 tested cases (15%) [43,52]. Other studies have also associated *MYC* abnormalities with disease progression, extramedullary disease, or plasmablastic transformation in myeloma [52,53,54]. Given the high overall incidence of MYC overexpression in P/A-MM, this marker lacks sufficient specificity for the differential diagnosis between P/A-MM and PBL [55,56]. Regarding plasmacytoma, although studies have observed that EBV-positive extramedullary plasmacytoma can be accompanied by MYC overexpression and plasmablastic/anaplastic morphology [30,57], the direct correlation between MYC overexpression and plasmablastic morphology remains to be further confirmed due to the limited sample size of such cases.

Tumor-cell EBER status and systemic EBV infection:

In PBL, the diagnostically relevant finding is EBER within tumor cells demonstrated by in situ hybridization. Reported tumor-cell EBER frequencies vary with immune status, geography, case selection, and testing methods [58]. For example, EBER was detected in 22 of 47 tested cases (46.8%) in a 60-case HIV-negative Chinese series [25] and in 18 of 30 tested cases (60%) in a selected 34-case molecular cohort [39]. Tumor-cell EBER positivity can also occur in plasma cell neoplasms: comparative and regional cohorts have described 19 EBV-positive plasmacytomas and 15 EBV-positive plasma cell neoplasms, respectively [30,44]. By contrast, a meta-analysis associating active systemic EBV infection with MM risk addresses a different biological and diagnostic question and cannot establish tumor-cell EBER positivity or EBV-driven plasmablastic transformation [59]. Observed geographic differences should therefore be treated as hypothesis-generating. They may reflect population characteristics, immune status, case selection, morphologic definitions, or EBV detection methods rather than a proven difference in biological mechanisms.

Ki-67 proliferation index:

PBL commonly has a Ki-67 proliferation index of at least 70% and often above 90% [28]. Plasma cell neoplasms with plasmablastic or anaplastic morphology also show high and variable indices, including values above 90% in selected small studies and case reports [34,60,61,62,63,64,65]. The index differs between myeloma and plasmacytoma [30,33,64]. Because the distributions overlap and the evidence is heterogeneous, no Ki-67 cutoff reliably distinguishes the two categories.

T-cell markers:

PBL can aberrantly express markers such as CD2, CD3, CD4, and CD7 [66]. Aberrant CD3 is typically diffuse with a cytoplasmic staining pattern; CD4 co-expression is extremely rare [67]. P/APC-N may also show aberrant CD3 and CD4 expression [55,68,69].

Diagnostic pitfalls include overinterpreting tumor-cell EBER or MYC results. Tumor-cell EBER positivity supports the presence of PBL in the appropriate context but is not specific; an EBER-negative result does not exclude PBL [25,30,39,44,58]. Systemic EBV infection or reactivation must not be equated with tumor-cell EBER positivity. Similarly, MYC protein overexpression does not confirm a *MYC* rearrangement, and neither finding is specific to PBL or to a plasma cell neoplasm [43,52,55,56]. Discordant results should prompt review of specimen adequacy, the analytical method, and the broader clinicopathologic and genomic context.

## 5. Cytogenetic and Molecular Features

### 5.1. Cytogenetic Features

Cytogenetic alterations in plasma cell neoplasms:

Genetic abnormalities in MM can be broadly classified into primary abnormalities, occurring in the early stages of the disease, and secondary abnormalities acquired during disease progression. Primary abnormalities predominantly include structural variations involving the immunoglobulin heavy chain locus (*IGH*) and/or hyperdiploidy characterized by trisomies of multiple chromosomes. Recurrent myeloma-associated primary abnormalities include *IGH* translocations such as t(11;14)(q13;q32) *CCND1/IGH* and t(6;14)(p21;q32) *CCND3/IGH*, as well as hyperdiploidy. High-risk abnormalities include t(4;14)(p16.3;q32) *FGFR3/MMSET/IGH*, t(14;16)(q32;q23) *IGH/MAF*, t(14;20)(q32;q12) *IGH/MAFB* [70].

Secondary cytogenetic abnormalities acquired during myeloma progression include 1q gain or amplification, del(17p), del(1p), and increasingly complex structural variants involving *MYC* [71]. Chromothripsis, templated insertions, and chromoplexy are also described in advanced disease [71,72]. Reported frequencies vary substantially across cohorts and should be linked to the specific plasma cell neoplasm subtype and testing platform rather than generalized to all P/APC-N.

SPB may exhibit hyperdiploidy and chromosomal translocations common in MM, including t(11;14), t(4;14), t(14;16), t(14;20), t(6;14), and t(8;14). High-risk karyotypic abnormalities occur in a minority of cases with poor prognosis, including biallelic del(1p32), gain(1q), and del(17p) [73].

Definitions of ploidy and adverse cytogenetic groups differ among studies, and individual cohorts have used different combinations of conventional karyotyping and FISH [70,71,74]. These methodological differences limit direct comparison of reported frequencies and should be stated when molecular findings are used diagnostically.

Cytogenetic features of P/APC-N:

The best-characterized molecular data concern P/A-MM; evidence for plasmablastic or anaplastic plasmacytoma is more limited. These tumors may retain primary myeloma-associated abnormalities while acquiring secondary high-risk lesions during progression. P/APC-N typically retains early primary abnormalities of MM, such as hyperdiploidy (trisomies of odd-numbered chromosomes 3, 5, 9, 11, 15, 19, and 21) and *IGH* translocations (e.g., t(11;14), t(4;14) and t(14;16)) [21,43,75,76,77,78,79].

Morphologic progression in P/A-MM is frequently accompanied by secondary high-risk alterations, including 1q21 gain or amplification, del(17p), del(1p), and chromosome 13 abnormalities [16,34,43,53,55,74,77,78,80,81,82,83,84,85,86,87,88].

*MYC* structural abnormalities may also emerge during progression, particularly with plasmablastic transformation or extramedullary disease [30,43,52,53,54].

Cytogenetic features of PBL:

*MYC* rearrangement is recurrent but heterogeneous in PBL, although its reported frequency varies substantially between cohorts. WHO-HAEM5–related summaries report *MYC* translocations in approximately half of cases, whereas a selected 34-case molecular cohort detected a *MYC* rearrangement in 26 of 30 tested tumors (87%) [39,89]. This difference illustrates the effects of case selection and testing method; neither estimate constitutes a diagnostic threshold.

An early array comparative genomic hybridization study reported that the copy-number profile of PBL correlated more closely with DLBCL than with plasma cell myeloma, with approximate Pearson correlation coefficients of 0.63 and 0.10, respectively [90]. These values describe cohort-level similarities in copy-number alterations and should not be extrapolated to gene-expression profiles, somatic mutation patterns, or individual-case classification.

Hyperdiploidy (particularly trisomies of chromosomes 3, 5, 9, 11, 15, 19, and 21) is a hallmark feature of MM and its advanced-stage P/APC-N, whereas it typically does not occur in PBL. Although both entities may show 1q gain or trisomy 7, neither finding is independently discriminatory [39].

The same early array comparative genomic hybridization study included 16 PBL and 8 plasma cell myeloma cases. Gain of 16p13.3 was detected in 6 of 16 PBL cases and 0 of 8 myeloma cases, with FISH validation performed in still smaller subsets [90]. Although potentially informative, this single small study has not established 16p13.3 gain as a validated discriminator for individual cases.

A *MYC* rearrangement is recurrent in PBL but is not specific. Plasma cell neoplasms can acquire *MYC* rearrangements or other structural abnormalities during progression, particularly with plasmablastic transformation or extramedullary disease [30,43,52,53,54]. Therefore, no isolated *MYC* rearrangement can distinguish PBL from a plasmablastic or anaplastic plasma cell neoplasm.

### 5.2. Molecular Features

Molecular alterations in plasma cell neoplasms and P/APC-N:

In addition to cytogenetic abnormalities, plasma cell neoplasms may acquire recurrent gene-level alterations during disease progression. *TP53* loss or mutation may accompany del(17p), and mutations involving signaling pathways, particularly the RAS/MAPK pathway, are common in advanced disease.

Recent research has identified *KRAS* and *TP53* as independent prognostic factors for SPB progression; combining high-risk karyotypes with *KRAS* and *TP53* defines prognosis more accurately [73].

In cases of plasma cell neoplasms with extramedullary involvement or discordant transformation, tumor clones frequently acquire genomic profiles of higher complexity and exhibit high-frequency mutations in the RAS pathway (e.g., *KRAS*, *BRAF* V600E) [53,75,87,91].

Molecular characteristics of PBL:

The molecular landscape of PBL is heterogeneous and should be described according to the analytical platform used.

Gene-expression studies have demonstrated downregulation of B-cell receptor signaling and activation of terminal B-cell and plasma-cell differentiation programs. At the transcriptional level, PBL therefore shows substantial biological overlap with plasmablasts and plasma-cell neoplasms, although the observed relationships depend on the comparator cohorts and analytical methods used [92].

Regarding the mutational profile, EBV-negative PBL cases exhibit higher chromosomal complexity, frequently harbor mutations in *PRDM1*/BLIMP1, *TP53*, and *CARD11*, as well as abnormalities in the MAPK/ERK pathway [39,89,93,94]. Conversely, *STAT3* gene mutations are more common in EBV-positive PBL cases [39,89].

*MYC* dysregulation in PBL should be interpreted at multiple molecular levels. MYC protein expression, gene rearrangement, copy-number alteration, and broader pathway dysregulation represent distinct biological findings and should be reported separately.

### 5.3. Integrated Cytogenetic and Molecular Findings in the Differential Diagnosis

Cytogenetic and molecular findings are most appropriately used as adjuncts in cases that remain unresolved after integration of the clinical context, morphology, immunophenotype, and tumor-cell EBER status. Most proposed genomic differences between P/APC-N and PBL were derived from relatively small or platform-specific cohorts and have not been validated as stand-alone classifiers for individual cases.

Among cytogenetic findings, hyperdiploidy and recurrent myeloma-associated *IGH* translocations support a plasma cell neoplasm lineage when present in the appropriate clinicopathologic context. In contrast, *MYC* rearrangements are recurrent in PBL but are not specific, because plasma cell neoplasms can acquire *MYC* rearrangements during progression, particularly in plasmablastic transformation or extramedullary disease [30,43,52,53,54].

At the molecular level, differences in recurrent mutations and signaling pathways may provide additional supportive information. However, mutations involving *TP53*, the RAS/MAPK pathway, and other oncogenic pathways may occur in both disease groups and should not be used in isolation for classification.

Accordingly, MYC protein expression, *MYC* rearrangement, *MYC* copy-number change, and broader MYC pathway dysregulation should be clearly distinguished. No isolated MYC-related result can distinguish PBL from a plasmablastic or anaplastic plasma cell neoplasm.

### 5.4. Recommended Detection Technologies

FISH should focus on detecting *MYC* and *IGH* gene rearrangements and screening for translocations characteristic of myeloma, such as *CCND1*, *FGFR3*, and *MAF*. For molecular characterization, targeted or comprehensive next-generation sequencing (NGS) may be used to detect recurrent mutations involving *TP53*, *KRAS*, *BRAF*, and other relevant pathways.

Because complex *MYC* structural variations, including nested inversions or non-Ig insertions, may be missed by conventional break-apart probes, NGS-based structural variant analysis or optical genome mapping (OGM) may be considered in challenging cases, particularly those with complex chromothripsis or other structural abnormalities [95,96,97].

## 6. Clinical Significance of the Differential Diagnosis

Correct classification has immediate clinical consequences because multiple myeloma, SPB, EMP, and PBL are staged, treated, and monitored within different disease frameworks. P/A-MM is managed within myeloma pathways, localized plasmacytoma requires assessment for local control and occult systemic disease, and PBL is managed as an aggressive large B-cell lymphoma. Misclassification can therefore alter systemic workup, treatment selection, response assessment, follow-up, and prognostic counseling [98,99,100,101,102,103,104,105,106,107]. The therapeutic contrast explains why an integrated diagnosis is necessary; it is not intended to provide regimen-level guidance. Evidence for plasmablastic variants is largely retrospective and heterogeneous, and direct comparisons between plasma cell neoplasm and PBL cohorts are not reliable. Entity-specific decisions regarding systemic therapy, radiotherapy, stem-cell transplantation, and novel agents should follow current disease-specific guidance and multidisciplinary review [98,99,100,101,102,103,104,105,106,107].

Published treatment-outcome data illustrate the clinical importance of classification but do not support direct cross-entity comparison. In a 344-patient multicenter PBL cohort, median progression-free survival was 1.4 years, and median overall survival was 5.0 years. EPOCH and CHOP or CHOP-like regimens produced similar overall response rates (64% versus 66%) and complete response rates (52% versus 52%); higher-intensity chemotherapy and frontline proteasome-inhibitor use were not associated with improved overall or progression-free survival in multivariable analyses [98]. In a single-center cohort of 67 patients with anaplastic MM, treatment data were available for 56 patients: the overall response rate was 76.8%, median progression-free survival was 6.0 months, and median overall survival was 29.5 months. Autologous stem cell transplantation was associated with longer progression-free and overall survival, although younger age and other baseline differences limit causal interpretation [27]. These studies used different eligibility criteria, host populations, and treatment eras. They support disease-specific treatment pathways but should not be read as a head-to-head comparison.

Cases that remain indeterminate after integrated clinicopathologic evaluation should undergo multidisciplinary review before entity-specific therapy is selected. Recommendations regarding individual regimens, drug doses, radiotherapy schedules, and transplantation are beyond the scope of this diagnostic review and should follow current disease-specific guidance and individualized clinical assessment.

## 7. Prognostic Features and Reported Survival Outcomes

Within plasma cell neoplasms, plasmablastic morphology has been associated with aggressive clinical behavior and adverse cytogenetic features in retrospective cohorts [27,81]. However, the available studies are heterogeneous and include small case series, selected institutional cohorts, and variably defined morphologic categories. Their survival estimates should therefore not be treated as universally applicable.

A contemporary US multicenter retrospective study included 344 patients with PBL and reported a median overall survival of 5.0 years, with outcomes differing among immune-status groups [98]. Notably, survival outcomes in PBL vary significantly depending on the patient’s underlying immune status. Patients with HIV infection typically exhibit the most favorable prognosis [108]. This survival advantage may be attributed to their generally younger age at diagnosis, their ability to restore immune surveillance through antiretroviral therapy, and the frequent association with EBV positivity. Conversely, patients with prior organ transplantation (PTLD-PBL) had the poorest prognosis, with a median OS of 1.1 years [98].

Reported survival outcomes should be interpreted in the context of disease stage, host immune status, treatment era, cohort selection, sample size, and diagnostic criteria. Accurate classification remains essential because prognosis and management must be estimated within the appropriate disease-specific framework rather than inferred from plasmablastic morphology alone.

## 8. Discussion

**Figure 3:**
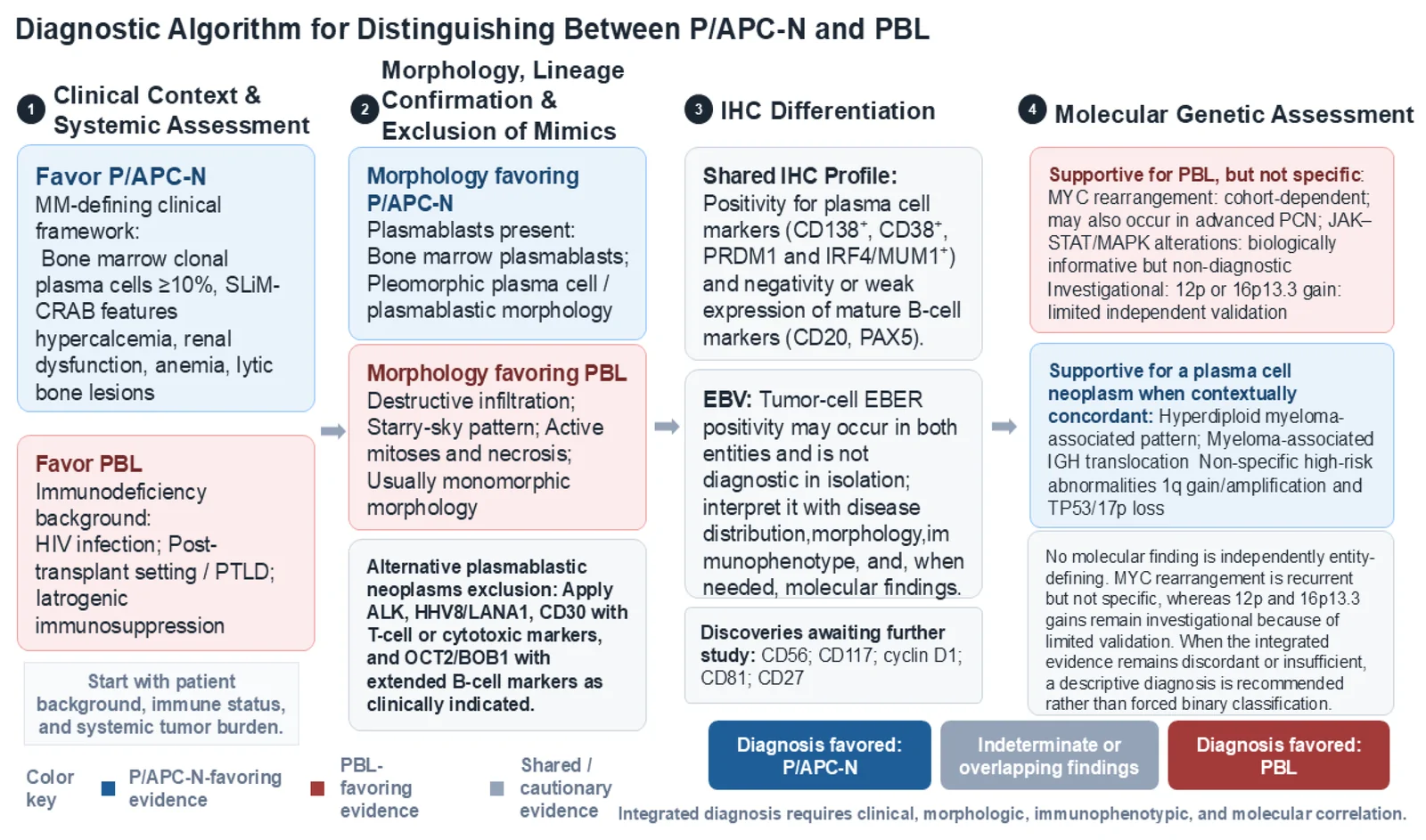
Proposed four-step diagnostic framework. Evaluation proceeds sequentially from clinical context and systemic disease assessment to morphologic and lineage confirmation, extended immunohistochemical and tumor-cell EBER evaluation, and, when necessary, molecular genetic testing. Blue boxes indicate findings favoring a plasmablastic/anaplastic plasma cell neoplasm or plasmablastic myeloma, red boxes indicate findings favoring plasmablastic lymphoma, and gray boxes represent shared or cautionary findings. Gray arrows indicate the sequence of the four diagnostic steps. This framework is synthesized from WHO-HAEM5 and published clinicopathologic, immunophenotypic, molecular, and biopsy-workup evidence [1,24,28,29,30,31,32,39,43,58,70,89,90].

Proposed Integrated Diagnostic Framework (Figure 3).

Step 1: Clinical background and systemic assessment. Evaluation should initiate with clinical history, immune status, disease distribution, marrow findings, serum and urine studies, and imaging.

Findings supporting a plasma cell neoplasm, particularly P/A-MM, include fulfillment of formal MM criteria: at least 10% clonal bone marrow plasma cells or a biopsy-proven bony or extramedullary plasmacytoma together with a myeloma-defining event, including SLiM-CRAB features. Features pointing to PBL: Presence of significant immunodeficiency, such as HIV infection (high positivity rate), PTLD, or iatrogenic immunosuppression.

Step 2: Morphology, lineage confirmation and exclusion of mimics.

Before proceeding to the P/APC-N versus PBL distinction, alternative neoplasms with plasmablastic morphology should be excluded using a context-directed panel. Diffuse or characteristic cytoplasmic ALK expression should prompt evaluation for ALK-positive large B-cell lymphoma and, when supported by the morphology and lineage findings, ALK-positive anaplastic large cell lymphoma. Tumor-cell HHV8/KSHV positivity should redirect the evaluation toward PEL, extracavitary PEL, or another HHV8-associated disease, with interpretation alongside other clinical findings. In EBER-positive cases with a retained mature B-cell program, EBV-positive DLBCL and other EBV-associated large B-cell lymphomas should be considered. OCT2, BOB1, and CD30 may assist with lineage assessment and differential diagnosis but are not independently entity-defining. If an alternative entity is supported, the case should exit the P/APC-N–PBL algorithm and be classified accordingly.

For suspected P/A-MM, bone marrow aspirate smears and tissue biopsies should be evaluated for the cytologic spectrum of mature plasma cells, immature plasma cells, plasmablasts, and anaplastic forms. Morphological diversity is observed, including plasmablasts, plasma cells, and anaplastic variants (occasionally with megakaryocytes). Plasmacytoma should be named separately when systemic MM criteria are not fulfilled. No validated universal plasmablast-percentage cutoff distinguishes a plasma cell neoplasm from PBL.

PBL usually shows a destructive, relatively monomorphic plasmablastic or immunoblastic infiltrate with frequent mitoses, apoptosis, and sometimes a starry-sky pattern or necrosis. Marked pleomorphism may raise consideration of an anaplastic plasma cell neoplasm, but these features overlap and cannot independently classify a case.

Step 3: Immunophenotypic and tumor-cell EBER assessment.

Both categories commonly express CD138, CD38, IRF4/MUM1, and PRDM1/BLIMP1 and show absent or weak CD20 and PAX5. Tumor-cell EBER positivity supports PBL in the appropriate clinicopathologic context but is not specific because rare plasma cell neoplasms are also EBER positive. Conversely, an EBER-negative result does not exclude PBL. CD56, cyclin D1, CD117, CD81, and CD27 may provide context, but available evidence is derived largely from small retrospective studies and case reports.

No single immunohistochemical or viral marker can definitively distinguish P/APC-N from PBL.

Step 4: Molecular assessment.

If a definitive diagnosis remains elusive through the aforementioned steps, chromosomal alterations should be detected using FISH, NGS, or OGM technologies.

Findings that may support PBL in the appropriate context include a *MYC* rearrangement and recurrent alterations involving the Janus kinase–signal transducer and activator of transcription (JAK–STAT) or mitogen-activated protein kinase (MAPK) pathways. These abnormalities are not specific, and their reported frequencies are cohort- and method-dependent. Gains involving 12p or 16p13.3 remain investigational and should not be used as independent diagnostic criteria.

Findings that may support a plasma cell neoplasm include a hyperdiploid myeloma-associated pattern or a recurrent myeloma-associated *IGH* translocation. Secondary high-risk abnormalities, including 1q gain or amplification, 17p/*TP53* loss, and *MYC* abnormalities, are not independently discriminatory.

If the evidence remains limited, discordant, or genuinely overlapping after all available studies, classification should not be forced on the basis of an isolated molecular finding. A descriptive diagnosis should summarize the evidence favoring each category, identify unresolved limitations, and recommend additional clinical evaluation, tissue sampling, or ancillary testing where appropriate.

## 9. Conclusions and Future Directions

The distinction between plasmablastic/anaplastic plasma cell neoplasms and plasmablastic lymphoma requires a stepwise, integrated approach. Clinical history, systemic disease distribution, and evidence of an underlying plasma cell neoplasm should first establish the diagnostic context. Morphology then defines the plasmablastic spectrum and guides the initial differential diagnosis, after which immunophenotypic and viral studies assess lineage and terminal B-cell/plasma-cell differentiation. Cytogenetic and molecular findings are most informative when applied to cases that remain ambiguous and when interpreted in conjunction with the preceding clinicopathologic evidence.

No single morphologic feature or ancillary marker can reliably distinguish all cases. Genuine overlap and diagnostically indeterminate cases therefore remain unavoidable, particularly when tissue is limited or different evidence domains are discordant. In such situations, classification should not be forced on the basis of an isolated result. Instead, the pathology report should summarize the evidence favoring each category, identify unresolved conflicts and specimen limitations, and recommend the additional clinical information, systemic evaluation, tissue sampling, or ancillary studies most likely to clarify the diagnosis.

Future studies should prioritize well-characterized, multi-institutional cohorts directly comparing these two disease categories using consistent diagnostic definitions and testing methods. Greater standardization of morphologic assessment, ancillary testing, and integrated reporting would facilitate comparison across studies and help determine the true discriminatory value of currently proposed features. The proposed diagnostic framework also requires validation in independent cohorts, particularly in cases with limited tissue, discordant findings, or overlapping clinicopathologic features. Such work may improve diagnostic reproducibility while providing a more evidence-based approach to the classification and reporting of genuine gray-zone cases.

## Figures and Tables

**Table 1:** Differential immunophenotypic features of plasmablastic/anaplastic plasma cell neoplasms (P/APC-N) and plasmablastic lymphoma (PBL). Representative denominators are provided when available and are not pooled estimates. Frequencies vary across cohorts, specimen types, case definitions, and detection methods. No single marker is independently diagnostic; all findings require integration with the clinical presentation, disease distribution, morphology, tumor-cell EBER status, and molecular findings.

Marker	P/APC-N	PBL	Diagnostic Value/Limitations
CD20, PAX5	Usually absent.	Usually absent or weakly expressed.	Loss of mature B-cell markers supports terminal differentiation but does not distinguish the two categories.
Plasma cell markers	CD38, CD138, IRF4/MUM1, and PRDM1/BLIMP1 are commonly expressed.	CD38, CD138, IRF4/MUM1, and PRDM1/BLIMP1 are commonly expressed.	These markers establish terminal B-cell/plasma-cell differentiation but have little independent discriminatory value.
Tumor-cell EBER by in situ hybridization	Usually absent. Positivity has been reported in a minority of cases and varies according to plasma cell neoplasm subtype, anatomic site, and population.	Positive in approximately 80% of cases, with a higher rate in HIV-positive patients.	Tumor-cell EBER positivity supports PBL in the appropriate context but is not specific. It must not be equated with systemic EBV infection, reactivation, or circulating EBV DNA.
Ki-67 proliferation index	Variable; values above 90% are reported, mainly in small series and case reports.	Usually high and commonly exceeds 70%. Values above 90% occur but are not required.	The distributions overlap substantially, and no validated cutoff separates the entities.
CD19	Usually absent; rare positive cases are reported.	Usually absent; rare weak expression is reported.	Low discriminatory value because both categories commonly lack CD19.
CD45	Usually absent or dim. Acquisition of CD45 was reported in two recurrent P/A-MM cases in a small series.	Variable and often dim; reported estimates differ according to cohort composition and immune context.	Limited discriminatory value; staining intensity, specimen type, and clinical context must be considered.
CD56	Variable. Expression is common in conventional plasma cell myeloma but may be lost in plasmablastic, anaplastic, or extramedullary disease.	Usually absent; reported positivity rates vary across cohorts.	Contextually supportive only. CD56 may be lost in extramedullary or high-grade plasma cell neoplasms, and either result can occur in PBL.
cyclin D1	Positive in 4 of 25 P/A-MM cases (16%) in one cohort; higher rates are reported in conventional MM cohorts.	Usually absent, although rare positive cases occur.	Positivity may support a myeloma-associated background when concordant with other findings, but sensitivity and specificity are insufficient for stand-alone use.
MYC protein expression and *MYC* rearrangement	In one P/A-MM cohort, MYC protein was expressed in 17 of 23 tested cases (74%), whereas a *MYC* rearrangement was detected in 2 of 13 tested cases (15%).	A *MYC* rearrangement was detected in 26 of 30 tested cases (87%) in a selected 34-case molecular cohort, whereas lower frequencies are reported in broader series.	MYC protein overexpression, *MYC* rearrangement, and broader *MYC* dysregulation are distinct. None is independently entity-defining.
T-cell markers	Aberrant CD3 or CD4 expression is described mainly in individual cases and small series.	Aberrant CD2, CD3, CD4, or CD7 expression is described in case reports and small series.	A diagnostic pitfall rather than a reliable discriminator; lineage must be established with a broader panel.

“CD, cluster of differentiation.”

## Data Availability

The original contributions presented in this study are included in the article. Further inquiries can be directed to the corresponding author.

## Abbreviations

The following abbreviations are used in this manuscript:

WHO-HAEM5	5th edition of the World Health Organization
WHO	World Health Organization
P/APC-N	p lasmablastic/anaplastic plasma cell neoplasm
PBL	plasmablastic lymphoma
EBER	Epstein–Barr virus-encoded RNA
EBV	Epstein–Barr virus
FISH	fluorescence in situ hybridization
NGS	next-generation sequencing
OGM	optical genome mapping
IGH	immunoglobulin heavy-chain locus
MM	multiple myeloma
SPB	solitary plasmacytoma of bone
EMP	extramedullary plasmacytoma
DLBCL	diffuse large B-cell lymphoma
HIV	human immunodeficiency virus
PEL	primary effusion lymphoma
P/A-MM	plasmablastic/anaplastic multiple myeloma
H&E	hematoxylin and eosin
*JAK–STAT*	Janus kinase–signal transducer and activator of transcription
*MAPK*	mitogen-activated protein kinase
OS	overall survival
PFS	progression-free survival
